# Nurse’s attunement to patient’s meaning in life - a qualitative study of experiences of Dutch adults ageing in place

**DOI:** 10.1186/s12912-020-00431-z

**Published:** 2020-05-18

**Authors:** Susan Hupkens, Marleen Goumans, Peter Derkx, Anja Machielse

**Affiliations:** 1 Research Centre Innovations in Care, Rotterdam University of Applied Sciences, Rochussenstraat 198, 3015 EK Rotterdam, The Netherlands; 2 University of Humanistic Studies, Utrecht, the Netherlands

**Keywords:** Meaning in life, Older adults, Patient’s perspective, Nurse-patient relationship, Home nursing, Quality of care, Well-being, Healthy ageing, Positive health, Ageing in place

## Abstract

**Background:**

Meaning in life (MiL) is considered to be an important part of health and is associated with many positive outcomes in older adults, such as quality of life and longevity. As health promotors, nurses may take patients’ MiL into account in the care process. There is a knowledge gap in terms of what constitutes good care in relation to older patients’ MiL, and what the benefits may be for patients when nursing is attuned to this aspect. The purpose of this study was to explore the experiences of home nursing older adults in relation to nurses’ attunement to MiL.

**Methods:**

Gadamerian hermeneutic phenomenological design with semi-structured interviews. Participants were 24 aged home nursing patients. A framework of care ethical evaluation was used in the analysis. Multiple dialogues enhanced understanding.

**Results:**

Patients did not expect nurses’ regard for their MiL. They rather expected ‘normal contact’ and adequate physical care. Nurses showed that they were open to patients’ MiL by being interested in the patient as a person and by being attentive to specific and hidden needs. Participants explained that the nurse’s behaviour upon arrival set the tone: they knew immediately if there was room for MiL or not. All participants had positive and negative experiences with nurses’ behaviour in relation to MiL. Valued nursing care included maintaining a long, kind and reciprocal relationship; doing what was needed; and skilled personalised care. Participants mentioned ‘special ones’: nurses who attuned to them in a special way and did more than expected. Benefits of care that was attuned to patients’ MiL were: experiencing a cheerful moment, feeling secure, feeling like a valuable person and having a good day. Older adults also stressed that consideration for MiL helps identify what is important in healthcare.

**Conclusion:**

Aged homecare patients value nurses’ attunement to their MiL positively. Although patients regard MiL mostly as their own quest, nurses play a modest yet important role. Managers and educators should support nurses’ investment in reciprocal nurse-patient relationships.

## Background

Because of ageing populations worldwide [1], nurses’ patients increasingly consist of aged persons. Healthy ageing is considered to be an important objective for both nurses and patients [2]. What ‘health’ means depends on one’s definition [3, 4]. ‘Positive health’ is regarded as most relevant in the care for chronically ill (older) patients due to its holistic and subjectivist character [3]. Positive health is described as an individual multidimensional process that focusses on positive outcomes in order to enable adaptation to life’s challenges [5–8]. Huber included six dimensions of positive health in her model: bodily functions, mental functions & perceptions, quality of life, social & societal participation, daily functioning, and MiL & spirituality [5]. She regarded MiL as the most important dimension [9]. As suggested by adherents of positive health, research confirms that high levels of MiL in aged adults are associated with a higher quality of life [10], a healthier lifestyle [11], longevity [12–14] and a lower prevalence of age-related conditions [13, 15, 16]. In this article we focus exclusively on MiL. Human beings desire to live meaningful lives [17–19]. MiL is a personal perception or understanding about one’s life and activities and the value ascribed to them [20]. MiL encompasses both the ‘big questions in life’ (existential meaning) and the meaning of experiences on a daily basis (daily meaning) [21, 22]. Nurses have a fundamental responsibility to promote and restore health [23]. Since MiL is an important part of health, nurses may take patients’ MiL into account in the care process. Patients regard MiL as important and believe that health professionals, including nurses, can play a role [24]. What this role should be and what the patient’s benefits are, is scarcely discussed in empirical literature. Research in nursing homes shows that nurses play a role in patients’ MiL by taking care of their physical and mental well-being, by promoting cherished activities [25], and by a confirming and kind relationship that includes careful listening and respect for the patient as a person [26].

A limitation of the few available studies is that they all have been conducted in nursing homes, whereas nowadays most people age in place [2], steadily using more home nursing services [27]. In the Netherlands, where this study was conducted, 94% of persons over 65 age in place [28]. People ageing in their own homes could provide a different perspective on care in relation to MiL than those living in nursing homes, as living at home is an important source of MiL among older adults [29, 30]. There is a knowledge gap on the subject of home nurses’ recognising and responding to older patients’ MiL. Furthermore, the possible benefits of this care for patients are unclear.

Because MiL is different for every individual [19, 29] and older adults have different strategies to retain MiL [29, 31, 32], good care with respect to MiL requires individual attunement. We chose Tronto’s four elements of good care as a theoretical lens, as they clearly include this individual attunement. The elements are [33]:

- *Attentiveness:* Recognising the needs of the other. Attentiveness requires suspending one’s opinion or goals; it is concerned with the perspective of the other.
- *Responsibility:* Includes responsibility of many persons in society and is rooted in one’s cultural role: What can we do for the other from our position?
- *Competence:* This is related to practical caregiving. If care is not provided adequately and tailored to the individual, it can never be good.
- *Responsiveness:* Engagement with the position of the other (the patient) as he or she expresses it. In other words, does the care feel good from the patient’s standpoint [34]?

To understand what good care is in relation to a patient’s MiL, we clearly need insight into the patient’s perspective. Hence the aim of this study was to explore the experiences of older adults who receive home nursing, in terms of nurses’ attunement to patients’ MiL. Research questions were:

- What do older adults who receive home nursing expect and value from nurses regarding attunement of care to their MiL?
- What is the consequence of this care for the older adults?

## Methods

### Setting

Setting for the study was a large care provider in the metropolitan area of Rotterdam, a large multicultural city in the Netherlands. Home nursing in the Netherlands is provided by neighbourhood-based teams consisting of one or two registered nurses and 10–18 nursing assistants of various educational levels (in this paper all referred to as ‘nurses’ and ‘she’). They work in shifts. Nurses of the care provider noticed that a growing number of home nursing patients confronted them with their MiL issues, which nurses found difficult to respond to. During the research period nursing teams followed a training and coaching programme on MiL. At the start of the programme patients from four teams were asked to participate in this study in order to explore their experiences with nurses’ attunement to patients’ MiL. In the research period the care provider went through several organisational transitions, which resulted in numerous changes in nursing personnel and modifications of many procedures.

### Design

We chose a Gadamerian hermeneutic phenomenological approach for this study. This includes exploring the lifeworld of the participants through opening up, questioning and dialogue in order to arrive at a shared understanding, a ‘fusion of horizons’ [35–38].

### Participants

We asked nurses of three home nursing teams to find 4–8 patients who reflected the diversity in their neighbourhoods in terms of age, gender, cultural background, socio-economic status and health. When data saturation was approached, we selected a last fourth team to include patients, resulting in 24 participants. Mean age of participants was 82.3 (median 85). Most of them rated their health as moderate and lived alone (*n* = 18), were women (*n* = 18) and had a Dutch cultural background (*n* = 18). Sixteen participants had a religion, but five of them were no longer practising (Table 1).

**Table 1:**
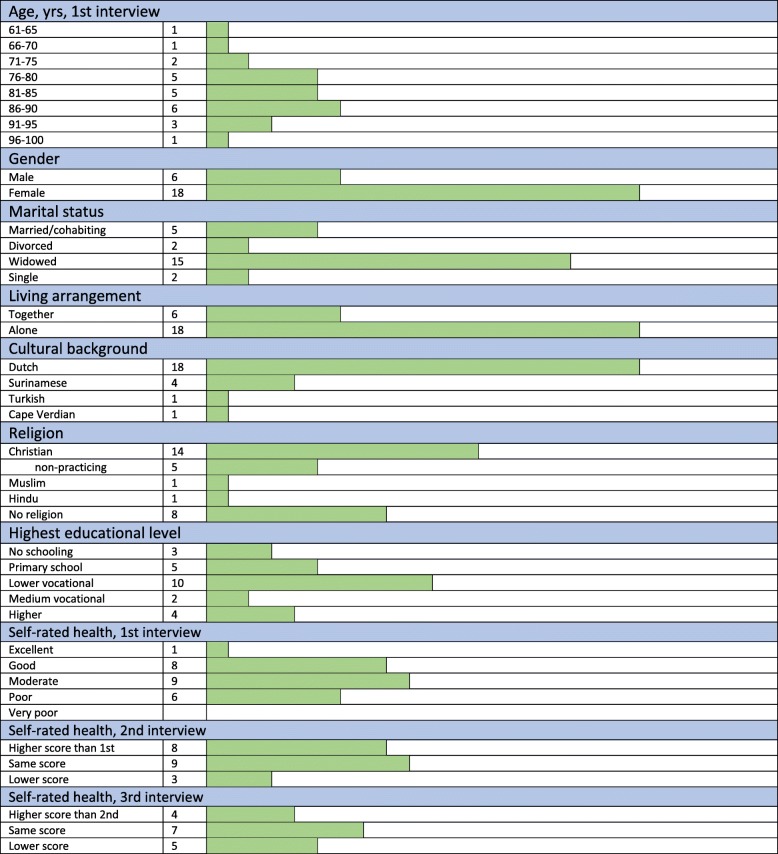
Background of participants

### Interviews

The main researcher (SH) interviewed participants three times, with 5–7-month intervals, between November 2015 and July 2018 in their homes. The aim of repeating interviews was mainly to arrive at a deeper understanding about our research topic. An interview guide was developed specifically for this research project (see Additional file 1). The semi-structured interviews consisted mainly of open questions, focussing on participants’ experiences [39, 40]. After ten interviews we evaluated the interview questions and made minor changes in formulation. In the interviews we firstly invited the aged person to share experiences on their MiL. These findings are reported in a separate article [29]. We subsequently asked the respondents about experiences with nurses’ attunement to MiL. Main questions were: Can you tell me something about your relationship with nurses? Do you think/notice that nurses are aware of your MiL? Can you tell me what you expect/value in this respect? Can you tell a recent example in which the nurse was attuned to your MiL? What was the consequence for you? We followed the flow of conversation and asked follow-up questions to explore the experience in more depth. Additional background information of participants was gathered (Table 1). For reasons of transferability, we gathered relevant background information of participants in the first interview. We chose items that, according to large-scale research, are associated with MiL [41–43]. Self-rated health was repeated in the successive interviews because this fluctuates in later life. Mean duration of interviews was 61 min (range: 32–112). In most interviews interviewer and participant were the only ones in attendance. Two couples, both partners clients of the homecare organisation, were interviewed together. Three participants were assisted by (translating) family members during the interviews because their Dutch language proficiency was limited. Most participants were interviewed three times (*n* = 16), four twice, and four once. Reasons for drop-out were deteriorating health (*n* = 3), death (*n* = 1), moving to a nursing home (*n* = 2) and ‘having nothing more to add’ (*n* = 2).

### Data analysis

Interviews were transcribed verbatim. In the analysing process we followed the steps of interpretative phenomenological analysis [40]. Data were analysed at two levels: firstly at an individual level and subsequently at an overarching level. At the individual level all interviews of each participant were analysed to arrive at a broad and deep understanding of the participant’s unique experiences in context. Next, overarching themes were interpreted for all data. This movement from the parts (individual) to the whole and vice versa [36–38] was repeated several times.

In Gadamerian hermeneutic phenomenology interpretation of data is characterised as a dialogue with the texts [36, 37, 44]. To analyse the content of our data we used a modified framework of care ethical evaluation of Kuis & Goossensen [34], which served as a ‘dialogue guide’ in this process and as such helped us to arrive at a shared understanding, a ‘fusion of horizons’. Both our study and care ethical evaluation intend to explore care from the perspective of patients and are inspired by Tronto’s four moral dimensions of care [34]. The (modified) framework consists of four main aspects:

1. What is at stake for the aged person?

- What are MiL sources for the aged person?
- How does the person retain MiL?
- What does he/she expect from the nurse?

2. Does the nurse recognise the person’s MiL (and the way he/she deals with it)?
3. How does the nurse respond to the patient (attunement to MiL)?

- … to the struggle, concern, vulnerability, need or pain of the aged person?
- … to the strength and resilience of the aged person?

4. Does the care well to the patient?

- What is the consequence of that?

In accordance with our research questions this article focusses on aspects 1c-4a. Findings of aspects 1a and 1b are reported in a separate article [29], but are included in the analysis of the interviews at the individual level (see Additional file 2, 3, 4, 5, 6 and 7). Initial analysis was done by the main researcher (SH). To further develop understanding as a ‘fusion of horizons’, dialogue was part of all phases of data analysis. In a dialogue, through open questioning and answering, an understanding emerges which transcends the subjective opinions of the participants [36, 37]. Firstly, dialogues about interpretations were part of the successive interviews with older adults. Secondly, dialogues took place in two research groups: the first one was based within the homecare organisation and consisted of patients, nurses, spiritual counsellors and researchers (MG and SH), the second research group was university-based and consisted of a nurse/health scientist (SH), a health scientist (MG), a philosopher (PD) and a philosopher/social scientist (AM). Pre-understanding of researchers derived from professional work in nursing, philosophy, research, reading, and personal experiences with ageing dear ones.

### Rigour

Multiple interviews per participant and continuing dialogue promoted credibility of this study [39, 44]. Dependability and confirmability were established by recorded and verbatim-transcribed data; analytical software (Atlas-ti 6.2.28); and analytical steps and an analytical framework [40, 45]. Reflexivity was fostered through a research diary and dialogues [38, 44]. In reporting this article we follow the COREQ [46].

## Results

### Good care in relation to MiL at two levels

Findings have meaning first of all at the individual level in their own context. We therefore provide six examples as results of the individual analysis, structured by modified framework of care ethical evaluation (Additional file 2, 3, 4, 5, 6 and 7). To enable the reader to understand the findings in context, the examples include all aspects of this framework. The examples are chosen from the four neighbourhoods (A-D) and display the diverse backgrounds of our participants (see Table 1).

Next, we present our findings at the overarching level structured by analytical aspects and themes (see Table 2: Themes). The background of participants is summarised in Table 1.

**Table 2:** Themes

Analytical questions	Themes
**Expectations**	‘Simply normal contact’, don’t expect consideration for MiL from nurses
What does the person expect from the nurse?	
	Adequate physical care, no MiL support
**Recognising MiL**	Setting the tone
Does the nurse recognize the person’s MiL (and the way he/she deals with it)?	Showing interest in the person
	Being attentive to specific and hidden needs of patients
**Response**	Maintaining a long, kind and reciprocal relationship
How does the nurse respond to the patient (attunement to MiL)?	
	Doing what is needed
	Skilled personalised care
	The special ones
**Consequence**	A cheerful moment that lifts me up or a superficial encounter
Does the care offered do well to the patient? What is the consequence of that?	
	Feeling secure or insecure
	Feeling like a valuable equal person, a dependent patient, or the nurse’s coach
	Having a good day thanks to good humane care, or suffering due to bad care
	Emphasising what is important in healthcare

### Expectations of older persons

Participants shared their expectations with us regarding their contact with nurses and the provided care in relation to their MiL.

#### ‘Simply normal contact’, don’t expect consideration for MiL from nurses

Participants expressed that they mainly expect ‘normal contact’. When we asked what ‘normal contact’ was for them, they mentioned many expectations about nurses’ attitudes. They expected nurses to be friendly and polite. Several participants reported experiences of nurses being impolite or rude. They wanted to be treated like a competent grown-up and not as a ‘demented granny’. Participants expected nurses to be discreet and not impertinent, for instance by not looking in cupboards unasked. Participants expected nurses to meet their duty to arrive on time and provide care as agreed.

*I expect simply normal contact, just being kind to each other. (C1.1, age 86-90)*

Many participants told us that the contact with nurses is superficial. They said they don’t expect nurses to have regard for Mil and seldom share MiL issues with them. They preferred to share this with near ones or keep these issues to themselves, for they experience MiL as something they must achieve by themselves. They do not want to bother others with it.

*I don’t think they [nurses] know what is important to me. We talk about normal superficial subjects like children, holidays. Things about meaning in life are mine. If I shared them with anyone, it would be with my son or other family members and not with the nurses. They go from one patient to the other and in the end, I am not more than a number to them. (D2.2, age 96-100)*

*I told them about the loss of my last friends … eh … and they sympathised with me, but you know, they have their own family. So, I keep it as much as possible to myself. You know, you shouldn’t bother others with your grief and worries. You just shouldn’t. (A3.3, age 76-80)*

#### Adequate physical care, no MiL support

Many participants believed that nursing is limited to physical care. First of all, they expected nurses to provide this care adequately and with technical proficiency.

*They are there for their work. They help me with taking a shower; they dry and rub me with body lotion. They even dry the shower stall. (A3.2, age 76-80)*

Some participants expressed that they don’t believe nurses are competent to provide support in MiL, but most of all they experienced that nurses lack the time, knowledge or attitude.

*I am expecting practical things from them, like fastening a button. To ask: ‘Can I mean something for you?’ … They can mainly do something for me. ‘To mean something’ is deeper. Then you must sit down, stay seated and listen. (D5.3, age 96-100)*

### Recognising what is at stake

Although most participants did not expect nurses to take MiL into consideration in their care, they nonetheless shared several examples with us showing that nurses were open to patients’ MiL. Participants also provided examples where nurses neglected MiL.

#### Setting the tone

In our dialogues the older adults explained that nurses already set the tone for the encounter when entering the patient’s house. Participants said they immediately notice whether the nurse is in a good or a bad mood, when there is something bothering her, if she is in a hurry. They explained that they adapt to this condition of the nurse. For some participants the encounter had a large impact on their day, for others this was less important.

*They are like the weather: When they are in a bad mood, they are unable to enter joyfully. And I won’t react too much. But if they enter with good cheer, it gives me a boost like: Cheer-up! Let’s go for it! (D4.1, age 76-80)*

The nurse’s behaviour upon arriving also sets the tone for space for MiL. Many participants did not experience this space with nurses because of their time slots and their task-oriented behaviour.

*Sometimes the nurse enters and from the hall she yells: ‘How are you?’ And I am here. Then she throws down her coat and focusses on the book (patient file). I don’t know what to answer then. ‘How are you’ is a big question. But if it is asked in such a way, I cannot respond. Yes, if you sit down and ask me while you sit close to me … . (B1.1, age 86-90)*

#### Showing interest in the person

Participants appreciated that most nurses show interest in them as a person. Nurses asked, for instance, if they slept well or about the plans for the day. Participants told that nurses sometimes have time for a short talk or a cup of coffee, although this had become rare after organisational changes by the home care provider. Although they considered most talks to be superficial, without touching upon their MiL, they explained that it was nevertheless important for them that nurses be interested in them as whole persons.

*There are nurses who come back to a conversation we had three weeks ago! Then I conclude: they listened to me with attention, they took the effort to remember it and continue the conversation. And then I feel very happy. (D4.2, age 76-80)*

And yet, participants also gave examples of nurses who seemed there only to carry out their technical tasks. They experienced this as denigrating.

*When I feel that they solely come to pour that drop into my eye and put on those elastic stockings, only for the bare fact of doing this, it feels denigrating to me. I would like them to approach me with a basic interest in me. (D4.3, age 76-80)*

#### Being attentive to specific and hidden needs of patients

Participants related experiences with nurses who noticed specific needs. Sometimes the older adults tried to hide their pain or sadness, but nurses who knew them for a long time immediately recognised the signs. Others told about situations they could not oversee, when a nurse understood perfectly what they needed.

*Only those from the regular group, the ones I know already for a very long time, they immediately see if something is wrong … they see it in the person. I cannot hide it from them. Especially [name] and [name] … [name] asked: what is wrong? And I said: nothing. And we sat chatting for a little while and, eh … she just knew anyway! (A2.2, age 61-65)*

By contrast, some participants mentioned situations when nurses were inattentive, sometimes failing to properly assess the needs of the patient or omitting to ask follow-up questions to learn more about their situation.

*They could keep their eyes and ears more open to the people in the neighbourhood. I think people show more than they notice. If you are telling something, they come up with a story that is ten times worse. And then I won’t tell it anymore … They could ask a bit deeper: what is it that isn't going well? It is this attentiveness that I’m missing. (A1.1, age 76-80)*

According to some participants, the organisation asks too much attention, which blurs the focus of nurses’ real work: the patient.

*The nurses are being jerked around. Those changes in the organisation are an excuse for other procedures here. And [name nurse] has to explain all that to us, in her free time. But that has nothing to do with us. That’s not our business. We listen to them, but it distracts from what they come for. But most of all, it limits the pleasure they have in their work. And that is important to us too. (B5.2, age 71-75)*

### Nurse’s response

Although most participants mentioned that they don’t expect nurses to have consideration for their MiL, they nevertheless reported many experiences of care which they considered to be attuned to it. They also shared examples of non-attuned care.

#### Maintaining a long, kind and reciprocal relationship

Participants expressed special affection towards nurses or permanent staff who had cared for them for a long time. They explained that knowing the nurses was important to them. Then both patient and nurse share other things, like experiences with dear ones, hobbies, etc. Participants enjoyed this immensely. In our dialogues with participants the reciprocal character of the nurse-patient relationship emerged as pivotal theme. Participants appreciated nurses sharing their own lives with them. Many participants were already aware of the reciprocal character of the relationship, others realised it during our dialogues. Participants told that, just as nurses do with them, they do their best to be friendly and interested in the nurse as a person. Instead of only receiving, participants took pleasure in giving nurses something in return. Some offered fruit or drinks, others gave little presents.

*Well, they see all those materials on my table, and they ask about it and say: ‘you have been making such nice things!’ And I give them away to them. [name] had had a grandson and I have a little basket with baby socks which I made. And I asked her to choose one for her grandson. And then later on she gave me a picture of the child with the socks. And that is so nice! (B3.1, age 81-85)*

*Sometimes when they have a free hour they come to my home. I tell them: come to me. I’ll make you tea, coffee, whatever you want. And then they eat their lunch sandwich here and I really enjoy that. Then you have different conversations. More about what’s on their mind. And they say to me: You are just like a mom to us. And then we’re joking around. (A3.3, age 76-80)*

Participants also told us that they functioned as a ‘sounding board’ for the nurses. They listened carefully to their worries and gave them advice.

*This morning [name] was here and she told me about the problems she had with her children. And I was able to give her some advice. She also tells her own stories and that’s fine with me. We have a good relationship and that is part of it. (A2.2, age 61-65)*

Participants felt sorry for the nurses’ poor working conditions. They tried to help them by doing as much as possible by themselves. They also refrained from asking for more time or attention. Many participants complained about the large number of temporary workers. They feel less at ease with them.

#### Doing what is needed

Participants especially valued nurses’ decisiveness. They told us about incidents when they genuinely were in trouble, due to extreme pain, sickness, exhaustion or an unforeseen situation. The nurse who visited them immediately understood the position of the patient (attentiveness) and acted adequately, for instance by calling the family doctor, arranging devices, providing the right physical care, or by sitting next to the patient and listening.

*There are a few women [nurses], especially [name], who I really trust. I know her from the very first day and she would take care of anything I needed, without me even telling or asking her. She just did it. She arranged the dial-a-bus, a shower chair, everything. (D1.1, age 86-90)*

#### Skilled personalised care

Although self-rated health of participants hardly changed in the three interviews (Table 1), many stories of participants revealed how their condition deteriorated. They explained that skilled personalised care is very important to them. They have their own habits and wishes, fitting with their values. Besides, adequate care means less pain and fatigue.

*They do their work well: fast and well … When they dress my wound, they are very careful not to hurt me. That’s humane. And they bind my slippers onto my feet, so bacteria don’t get into my wound, because I cannot see it. They are caring.’ (C6.2, age 81-85)*

Participants shared that they preferred to be cared for by nurses they already knew for a long time: those nurses knew exactly what to do and how to do it properly. Additionally, those nurses do not take over activities that participants can still do themselves, or exactly the opposite: nurses do take over extra activities on a bad day. Participants complained about the frequent interim personnel. Some of the temporary workers showed limited technical skills. Every detail had to be explained to them, exhausting patients and causing them distress.

*Those few nurses do the care well. My leg is extremely painful. If you touch it like that, it hurts already. And one of those temporary nurses, she didn’t know how to bandage, although I told her how. And yes, after a little while the bandages fell off. And the consequence was that my legs became thicker and even more painful during that day. (A2.3, age 61-65)*

Many participants complained about the fact that home nurses were rarely on time. Others expressed their appreciation for nurses who were on time, so they could for instance go to church, which was important for them.

#### ‘The special ones’

Almost every participant mentioned a favourite nurse, ‘a special one’. These were nurses with whom a special connection was felt; they were attuned in a special way to the personality and needs of the patient. Many of these nurses showed all the positive themes mentioned in sections 3.3 and 3.4, and more. According to our participants, these ‘special ones’ did something extra for patients, something that was not prescribed in the nursing plan, even against the rules of the organisation, but which was highly appreciated. We heard many stories of ‘special ones’. For instance, a favourite nurse took letters to the mailbox for a patient with limited mobility; came by in her private time to show her new-born baby; walked the dog for a sick patient; enjoyed and danced to music together.

*[name] is my darling. When she visits me and my son’s music plays, she stands here dancing and I say: Hey, there is Tina Turner again! And she jokes about my untidy hair. We make fun of each other. And I say to the Lord: You give me exactly the girl I need! (C3.2, age 76-80)*

### Consequences for the older person

Our participants explained how the care, which they considered to be attuned to their MiL, or the lack thereof, impacted their life. Participants also mentioned a consequence for healthcare service.

#### A cheerful moment that lifts me up or a superficial encounter

For most participants the visits of the home nurse were gleeful moments during the day, especially if the nurse was one of their favourites. They told that a pleasant visit of a nurse helps start the day joyfully, it breaks the day and as a consequence they feel uplifted.

*Sometimes I am alone all day and they come twice a day. Most times they are busy, but sometimes I offer them a cup of coffee and we have a little talk. It gives a pleasant atmosphere and provides me with a cosy feeling. (D5.3, age 96-100)*

*I feel happy when the nurse enters my home, even if she can only stay for five minutes. It is attention and I always say: for human beings attention is more important than food. And when they pass my window they always wave, and in fact that is already contact. Marvellous. (D3.3, age 91-95)*

As explained before by participants, temporary staff, or a negative tone when the nurse arrives, resulted in encounters that remained superficial.

#### Feeling secure or insecure

The friendly, reciprocal contact with nurses provided participants with a sense of security: a known, trusted person was watching over them.

*They come to look after me because I am very old and have nobody. They check on me. That feels safe. They sit here for a little while and they always say: it is so cosy with you. They can rest here for a little while. I can relate to them, start a light conversation with them, because I worked with people all my life. (B4.3, age 96-100)*

A few participants told about nurses who did not respect their privacy or even displayed intimidating behaviour, which resulted in feelings of stress and unsafety.

*The big man [male nurse] was standing in front of me and said: you can hire me privately and I will be on time. You can pay me directly. And I thought: If I don’t promise to hire him, he’ll hit me … That’s unseemly behaviour. I have been of service to others my entire life in my job. I think things are moving the wrong way with healthcare. (D6.2, age 86-90)*

#### Feeling like a valuable equal person, a dependent patient, or the nurse’s coach

Participants shared with us that the long reciprocal relationship with permanent staff nurses, particularly the special ones, provided them with a feeling of trust and equality. They explained that it is important for them to be regarded as equal human beings instead of dependent patients. Participants felt valued when nurses thanked them for listening.

*Yes, they are open to me, so nice. They don’t sit here like a nurse but more like a good acquaintance. That’s what I like so much … As a patient you can be pitiable and as a human being you just feel normal. That’s it: I don’t feel like a patient. I don’t want to. I just want to be human among other humans … There is one nurse who calls me her friend. That’s so nice. (D1.3, age 86-90)*

*I appreciate the trust she has in me. Because when she is asking me, she knows I have an honest opinion ... however, most times I am just listening to them. (A1.3, age 76-80)*

Although participants appreciated a reciprocal relationship with familiar nurses, for some of them the balance between giving and receiving was off: the attention they paid to nurses’ worries overshadowed their own problems***.***

*When they run into difficulties in their work, they come to me. [Tells an example of another patient.] And then they turn to me for advice. Honestly, that puts a burden on me, because I keep thinking about it … There is hardly any focus on me. Well, on the other hand, I don’t take the opportunity to tell about myself … (A1.3, age 76-80)*

#### Having a good day thanks to good humane care, or suffering due to bad care

Participants explained how skilled care has a large impact on their life. If physical care is done correctly it limits pain, suffering and exhaustion, leaving room for them to do what is important to them, like gardening or visiting family. The provided care is a prerequisite for having a good day, living their life as they want to.

*They [permanent staff] are good women. They know everything, I don’t have to explain, and they do their work very well and then it is not painful. I am not stressed anymore. I can sleep again and eat again. (C5.1, age 66-70)*

Waiting for the nurse for no reason feels pointless for participants and limits the activities of that day.

*We still have an active life. I do as much as I can by myself. I had to be in the hospital on time. The taxi will not wait. It intrudes in my life when they are too late. I was there sitting and waiting, and they even didn’t call to say they were late … I was used to care independently for myself and my partner all my life. And when they don’t come on time, I lose part of my life. We don’t blame those nurses we know. It’s taken away by the policy of an organisation. It makes me feel curtailed. (B5.2, age 70-75)*

#### Emphasising what is important in healthcare

Many participants considered healthcare services to be deteriorating. Participants stressed that nurses’ concern for patients’ MiL was not only important for them as individuals, but also for healthcare in general. They explained that the focus on patients’ MiL also restored attention to what’s really important in healthcare.

*Well, I think that the higher you come in the organisation, the less focus there is on this aspect [MiL] and on emotions. And that is important for the people who give those trainings: that these very tiny spiritual notes are most important in the big picture.’ (D4.3, age 76-80)*

## Discussion

The aim of this study was to explore the experiences of older adults who receive home nursing in terms of nurses’ attunement to patients’ MiL. To our knowledge, it is the first study on this subject from the perspective of adults ageing in place, which is the majority of ageing people [2, 28]. MiL is an important part of health [5] and is vital for healthy ageing [13–16]. The results of this study provide a valuable insight into good (and bad) nursing care in relation to patients’ MiL. In this section we discuss our results in the context of other, rather scant scholarly literature. Our findings reveal that older adults receiving home nursing did not expect nurses to pay specific attention to MiL. At the same time, all our participants shared with us good and bad experiences of care that they considered as attuned to their MiL (or not) and which impacted their life. Our reflections on the findings are structured by Tronto’s four dimensions of good care. We end with some remarks about the nurse-patient relationship in homecare, as our results clearly show that good care attuned to patients’ MiL is embedded in this relationship.

### Good care in relation to patients’ MiL

As our results reveal, both at the individual level (Additional file 2, 3, 4, 5, 6 and 7) and the overarching level (Themes), good care in relation to MiL covers all four moral dimensions of Tronto: attentiveness, competence, responsibility and responsiveness.

#### Attentivenes

On the one hand our participants related positive experiences with nurses who were interested in them as a person, took the time and were attentive to their – sometimes unuttered – needs. On the other hand, participants complained about nurses who disregarded basic polite behaviour, didn’t have time for them, and paid attention exclusively to technical interventions. Because our study is concerned with the *patient’s* perspective, we are unable to reveal what happened inside the *nurses’* minds and hearts – as one of our participants said, *‘I cannot know what they perceive.’* Klaver & Baart unruffled elements of attentiveness (in oncology nursing): attentiveness is not only perceiving something but also realising what one perceives (interpretation). Attentiveness is enhanced if nurses have space and time to pay attention [47], which they mostly lack according to our participants. This study adds that a longstanding relationship enhances attentiveness, since observations can be interpreted in the context of the patient’s life. Healthcare managers should therefore guarantee adequate space and time to enable nurses to invest in longstanding relationships. To our participants, attentiveness to MiL is not only important in the nurse-patient relationship but also at an institutional level, as awareness of MiL emphasises what is important in healthcare.

#### Competence

Participants were unanimous about the importance of skilled personalised care for their MiL because it limits pain, exhaustion and stress, which they regard as negative conditions for MiL. This was also described for nursing home patients [25]. Some respondents doubted whether nurses were competent to provide support with MiL.

#### Responsibility

This dimension of good care emerged at three levels in our results. Firstly, participants considered nurses to be responsible for providing technically skilled care, keeping their commitments, arriving on time, doing what was needed, and adjusting the care to the specific needs and wishes of the patient. Secondly, participants were very clear that nurses’ (poor) working conditions were the responsibility of the management of the healthcare organisation. They blamed management for the nurses’ lack of time and the discontinuity in personnel, which negatively influenced their life. Thirdly, participants stressed that they regarded MiL as their own responsibility. This seems to contradict studies in nursing homes and in the general population, where authors conclude that nurses have an important supportive role in patients’ MiL [24–26]. Our results seem to deviate from these conclusions (for home nursing). Our participants emphasised that finding MiL is predominantly their own quest, although they sometimes shared MiL issues with family or friends. At first glance they expected ‘normal’ contact and adequate physical care from the nurses but no specific MiL support. Nonetheless, participants showed in their examples that through a kind equal and reciprocal nurse-patient relationship and skilled personalised care, nurses do support them: they enhance (or deteriorate) the conditions under which patients themselves maintain or find MiL. In other words, by attuning to patients’ MiL in their ‘normal’ daily behaviour nurses can support them in maintaining their MiL. We should not overestimate this role of the nurse though: the examples in the appendices of this article and earlier literature [42, 48–50] show that other circumstances play an important role regarding MiL in old age, such as deteriorating health, loss of dear ones, and other (negative or positive) life events. Compared to those conditions, nurses’ role may be modest yet remains important. The role of nurses is especially promising because home nurses have many opportunities during daily care to attune to patients’ MiL [51]. We therefore confirm that nurses have an important supportive role, as described in other papers [24–26], but our study adds that this role should be considered in the context of many other influencing circumstances. What’s more, recent healthcare interest in patients’ MiL should not lead to taking over patients’ responsibility through a formal approach – a ‘registering eye’, as Martinson described it [52]. Rather, professionals should adopt a modest caring role. By creating openness and space, allowing both personal closeness and professional (more distant) understanding, nurses can be present in the patient’s world yet without possessing it [52].

#### Responsiveness

Although participants did not expect nurses to attune care to their MiL, they were all able to provide positive examples of this behaviour. Care that was attuned to patients’ MiL felt good and participants benefitted from it: they were lifted up by a cheerful moment, they felt secure and valuable as persons instead of as patients, and had a good day thanks to good humane care.

### Reciprocal nurse-patient relationship in homecare as a vehicle for good care

As our results show, participants highly valued long, kind and reciprocal relationships with nurses. Attunement to patients’ MiL was embedded in this nurse-patient relationship. Literature supports centrality of the nurse-patient relationship in homecare, as our study did. With familiar and trusted nurses patients feel at ease, accepted and connected; those nurses provide physical care that is adjusted to the person; patients feel known as equal, valued individuals and are encouraged and motivated [53, 54]. Our participants emphasised that especially feeling as an equal person, instead of a patient, was important to them. The nurse-patient relationship provided them with the opportunity to enact favourite (social) roles and use their character strengths, which are pivotal for MiL [29, 55]. In a reciprocal relationship both partners give and receive. Although patients experienced benefits from giving something in return to nurses, this gradually became a burden for a few of our participants when their support of nurses’ problems started to overshadow their own. In reciprocal relationships, balancing giving and receiving is a continuous endeavour that affects both partners – in this case patients and nurses.

### Methodological considerations

The Gadamerian hermeneutic phenomenological approach enabled us to arrive at a mutual understanding of this subject, together with the participants. Multiple interviews with participants deepened understanding. Diverse backgrounds (of participants and researchers) and in-depth dialogues contributed to new insights, a ‘fusion of horizons’, for both researchers and participants [36]. Many participants greatly enjoyed our conversations, as they seldom had the opportunity to discuss topics beyond a superficial level. The theory of Tronto and the questions of care ethical evaluation proved to be a helpful framework to analyse nursing in relation to MiL.

Our study has limitations. Firstly, sampling and attrition limit the transferability of this research. Secondly, the credibility of three interviews was compromised by the presence and translation of family members of non-Dutch-speaking participants, even as this enabled us to include participants of more cultural backgrounds. Thirdly, considerable organisational transitions during the research period definitely impacted the results of this study. But healthcare is always changing anyway. The organisational turmoil revealed the important role that management plays in safeguarding conditions that promote – or inhibit – good care in relation to patients’ MiL. A last remark can be made regarding self-rated health: this measure failed to reflect the deteriorating physical condition of most of our participants, although their stories showed so. Others concluded before that older people tend to be over-optimistic in this measure [56, 57].

## Conclusion

In this study we explored what older adults who receive home nursing expect and value from nurses in terms of attunement of care to their MiL. We also investigated the benefits of this behaviour. We conclude that aged homecare patients value nurses’ attunement to their MiL positively. Although patients regard MiL mostly as their own quest, nurses play a modest yet important role. Benefits of this care for patients are experiencing a happy moment, feeling like a valuable person and having a good day. Besides, participants regard consideration for MiL as conducive to emphasising what is important in healthcare.

This article provides nurses with valuable knowledge and examples that may help them attune care to aged patients’ MiL. Nurses should have the opportunity to invest in reciprocal relationships with patients, which facilitates possibilities for MiL. Tronto’s four moral dimensions of good care could be a valuable framework for nurses to discuss good care in relation to patients’ MiL, as it was for our research. This paper may lead to healthcare management’s awareness of how staff discontinuity and lack of time negatively affects MiL of aged patients. Management should invest in an organisational culture that supports nurse-patient relationships. This paper (and the appendices) can serve as inspiration for nursing education. We hope it motivates nurse educators, both in schools and in practice, to facilitate reflective sessions on patients’ MiL and the role of the nurse.

## Supplementary information

**Additional file 1.** Interview guide

**Additional file 2.** Analysis of participant D4

**Additional file 3.** Analysis of participant A3

**Additional file 4.** Analysis of participant B4

**Additional file 5.** Analysis of participant C6

**Additional file 6.** Analysis of participant B5

**Additional file 7.** Analysis of participant C5

## Data Availability

The datasets generated and/or analysed during the current study are not publicly available, as ethical restrictions preclude the publication of raw data. Narratives with analytical questions and excerpts from the interviews are available in the additional files of this article.

## Abbreviation

MiL: Meaning in life
