# Supplementary material for: Nurse’s attunement to patient’s meaning in life - a qualitative study of experiences of Dutch adults ageing in place
Source: BMC Nurs. 2020 May 18;19:41. doi: 10.1186/s12912-020-00431-z (PMC7236336; doi:10.1186/s12912-020-00431-z)
Supplement: Supplementary file 1 — Additional file 1. Interview guide [file 12912_2020_431_MOESM1_ESM.pdf]

Interview guide

Topic	Interview 1	Interview 2	Interview 3
Introduction	Aim, information, informed consent	Aim, information, informed consent	Aim, information, informed consent
Background data	Age, gender, marital status, living arrangement, cultural background, religion, highest educational level, self-rated health	Self-rated health	Self-rated health
Current situation		How are you at the moment?<br>Important changes since last interview?	How are you at the moment?<br>Important changes since last interview?
MiL	Questions about respondent's MiL (*not this paper)*	Questions about respondent's MiL *(not this paper)*	Questions about respondent's MiL *(not this paper)*
Relationship with nurses	Can you tell me something about your relationship with nurses?	Can you tell me something about your relationship with nurses?<br><br>Last time you told .....<br>The same? / Changed? Example? Explain?	Last time you told .....<br>The same? / Changed?<br>Example? Explain?<br><br>Do I understand correctly that ....
Recognising MiL?	Do you feel that nurses are interested in you? How?<br><br>Do you think that nurses are aware of your MiL (or: what is important for you in life?) How do you notice this?	Do you feel that nurses are interested in you? How?<br><br>Do you think that nurses are aware of your MiL How do you notice this?<br><br>Last time you told .....<br>The same? / Changed?<br>Example? Explain?	Last time you told .....<br>The same? / Changed?<br>Example? Explain?<br><br>Do I understand correctly that ....
Expectations	Can you tell me what you expect in this regard? Competence?		

Attuning?	Can you tell a recent example in which the nurse was attuned to your MiL (to what is important for you in life)?<br>*(Ask details: Can you tell me more about it? What? Where? Who? How? Behaviour? Feeling? What mattered to you? Etc.)*<br><br>Ask for another example.	Can you tell a recent example in which the nurse was attuned to your MiL?<br>*(Ask details: Can you tell me more about it? What? Where? Who? How? Behaviour? Feeling? What mattered to you? Etc.)*<br><br>Last time you told .....<br>The same? / Changed?<br>Example? Explain?	Last time you told .....<br>The same? / Changed?<br>Example? Explain?<br><br>Do I understand correctly that ....
Value/ competence	Can you tell me what you value in this respect? Do you miss something? Competence?	Can you tell me what you value in this respect? Do you miss something? Competence?<br><br>Last time you told .....<br>The same? / Changed?<br>Example? Explain?	Last time you told .....<br>The same? / Changed?<br>Example? Explain?<br><br>Do I understand correctly that ....
Consequence	What was the consequence for you?	What was the consequence for you?<br><br>Last time you told .....<br>The same? / Changed?<br>Example? Explain?	Last time you told .....<br>The same / Changed?<br>Example? Explain?<br><br>Do I understand correctly that ....
Final question	Is there something you want to add regarding the subject of this conversation?	Is there something you want to add regarding the subject of this conversation?	Is there something you want to add regarding the subject of this conversation?
Finishing interview	Any questions?<br>Thank you	Any questions?<br>Thank you	Any questions?<br>Thank you
