# Supplementary material for: Nurse’s attunement to patient’s meaning in life - a qualitative study of experiences of Dutch adults ageing in place
Source: BMC Nurs. 2020 May 18;19:41. doi: 10.1186/s12912-020-00431-z (PMC7236336; doi:10.1186/s12912-020-00431-z)
Supplement: Supplementary file 2 — Additional file 2. Analysis of participant D4 [file 12912_2020_431_MOESM2_ESM.pdf]

Analytical questions	Participant D4 (age 76-80)
Introduction<br>1. What is at stake for the aged person?	Participant D4 lives in an apartment with a lovely terrace, covered with flowers. He lives alone since the death of his partner, years ago. He has five children. They maintain good relationships. Participant D4 suffers from severe progressive Parkinson's disease which limits his speech, movement and energy. He regularly feels sad about his deteriorating health condition. Participant D4 explains that meaning is *'like the weather: there are good and bad days.'*
a. What are MiL sources for the aged person?	1a. Participant D4's MiL sources are:<br>• Human relationships: the community in the apartment building, in church and his family. *'It is important to live in a community where one can count on one another.'*<br>• Nature. He loves to observe the birds in his aviary and care for the plants on his terrace. *'Life is so wonderful!'* Both nature and community connect him to the universe, to something bigger. For Paul this relates with (protestant) faith.<br>• Doing craftwork: making things. *'To do those pieces of work; that's my life.'*<br>(D4.1, D4.2, D4.3)
1b. How does the person retain MiL?	Participant D4 rarely talks about his worries regarding the deteriorating health condition and the future. He rather picks up some work to do. He tells that whenever he talks about it, he tries not to be pessimistic and to search for the humour in a situation. Participant D4 is a reflective person. He keeps pondering *'about the puzzle-pieces of life'* and also about the meaning of his actual life. Nature keeps him wondering: *'Enjoy the last sunbeam of the day.' 'That little bird can be sitting right next to your shoe!'* Furthermore, he tries to mean something for others, e.g. he takes care of the cat of his son now and then and visits sick neighbours. *'You have to be attentive to others.'* This also applies to the home nurses. He loves to ask them: '*Are you busy or do you want a coffee?'*<br>(D4.1, D4.2, D4.3)
1c. What does he/she expect from the nurse?	Participant D4 expects that nurses are well-trained, skilful and trustful. He expects them to keep to their agreements or, at least, call if this is not possible. He expects them to have some *'basic interest'* in him. *'When I feel that they solely come to pour that drop into my eye and put on those elastic stockings, only for the bare fact of doing this, it feels denigrating to me. I would like them to approach me with a basic interest in me.'* (D4.1, D4.2, D4.3)
2. Does the nurse recognize the person's MiL (and the way he/she deals with it)?	Participant D4 doubts this. Due to temporary staff he experiences most contact as superficial. On the other hand he tells about two nurses who do recognise it when he is feeling low. '*They have this personal attention.'* (D4.3)
3. How does the nurse respond to the patient (attunement to MiL)?	Participant D4 is annoyed by the fact that he never knows when the nurses come. *'I hate waiting, you know. It has a high impact on my day. I can read the newspaper but it feels obligatory. And then gradually the day is slipping away. And that makes me dissatisfied again because I wasted my time. I have suggested that they provide a prognosis regarding the estimated time they'll come but it had no result…'*<br>Although nurses come on time when he wants to go to church, he can

a. to the struggle, concern, vulnerability, need or pain of the aged person?	never be sure about appointments.<br>Participant D4 tells about the impact the nurses' mood has on him: *'They are like the weather: When they are in a bad mood they are unable to enter joyfully. And I won't react too much. But if they enter with good cheer, it gives me a boost like: Cheer-up! Let's go for it.'*<br><br>On bad days Participant D4 is a bit more silent: he doesn't want to transfer his bad mood to the nurses. A few nurses, who remarked changes in his condition, asked follow-up questions. Participant D4 suggests that they could do this more. Participant D4 tells that nurses take over a bit more on a bad day. He highly appreciates this.
b. to the strength and resilience of the aged person?	Participant D4 enjoys when the well-known nurses visit him, because they can talk about broader subjects, subjects that interest both, e.g. the birds, children, work. *'You become more interested in each other and that is reciprocal.'* He especially appreciates continuing a conversation. *'There are nurses who come back to a conversation we had three weeks ago! Then I conclude: they listened to me with attention, they took the effort to remember it and continue the conversation. And then I feel very happy.'*<br><br>When Participant D4 was visiting an ill neighbour, two nurses accidentally came by, while he was making tea for her. The relation between Participant D4 and the nurses has become more equal since then. He explains: *'That's what it is like when you are in care. You can encounter each other. And it doesn't matter whether you are caring or cared for.'*<br>(D4.1, D4.2, D4.3)
4. Does the care offered do well to the patient?	Yes and no.
a. If yes: what is the consequence?	Participant D4 emphasises that the physical help he receives is 'an *enormous support'* for him: *'They complement my handicaps which enables me to make something of my day.'* This especially valuable on a bad day: *'Taking over a bit more than usually then feels well for a human being.'*<br><br>He appreciates the reciprocal personal contact with nurses in long-standing relationships and especially the encounter as equal human beings. It feels good to be able to mean something for nurses as well.<br><br>Benefit for healthcare:<br>Participant D4 emphasises that attention for MiL is important in healthcare, not only for nursing but also for management. *'Well, I think that the higher you come in the organisation, the less focus there is on this aspect [MiL] and on emotions. And that is important for the people who give those trainings: that these very tiny spiritual notes are most important in the big picture.'*
b. If not: what is the consequence?	When the nurses are not coming on time the day slips away. When nurses are entering grumpy it has a negative impact on his mood.

	Participant D4 doesn't invest in the relationship with temporary staff. *'I can tell a lot of things but there is no consequence to it.'* (D4.1, D4.2, D4.3)
Additional remarks	Participant D4 loved to participate in the study. He was looking forward to our talks. He remarked that it contributed to his MiL: *'to participate in something that may bring forward something good.'* (D4.1, D4.3)
