# Supplementary material for: Nurse’s attunement to patient’s meaning in life - a qualitative study of experiences of Dutch adults ageing in place
Source: BMC Nurs. 2020 May 18;19:41. doi: 10.1186/s12912-020-00431-z (PMC7236336; doi:10.1186/s12912-020-00431-z)
Supplement: Supplementary file 3 — Additional file 3. Analysis of participant A3 [file 12912_2020_431_MOESM3_ESM.pdf]

**Analytical questions**	**Participant A3 (Age 76-80)**
Introduction<br>1. What is at stake for the aged person?<br><br><br><br><br><br><br><br><br><br>a. What are MiL sources for the aged person?	Participant A3 lives with his dog in the city centre in an old house. He tells me his life story. He divorced 16 years ago. He has no kids. Beside the marital problems, other family relations were troublesome as well. Participant A3 had a few good friends. However, between our first and second interview his last four friends, including his best friend, died. He says: *'Now I have nobody anymore, nobody who comes around here, nobody who puts her arms around me.'* Participant A3 had cancer some years ago. The cancer was cured but the therapies resulted in a fragile health status which limits his activities. Therefore home nurses assist Participant A3 with daily care.<br><br>Participant A3 wears a necklace with three pendants which reveal his most important MiL sources:<br>• A pendant with the emblem of his favourite soccer club. Participant A3 watches every match on television. It is a club which is supported by many working class people. Participant A3 emphasises: *'I am a normal person, a working- person'*. Participant A3 worked as a housekeeper till his retirement and he is proud of that.<br>• A pendant of his favourite animal, a dog. Participant A3's dog is his most important friend. *'He is my everything. He is my child, a prop and stay to me. He comes to me when I am sad. He makes me live. He is getting old. I always ask the Lord: Could I please stay a little longer, for him?'*<br>• The last pendant is an angel. It symbolises his faith (Roman Catholic). To live a morally good life is most important to him. *'Everything in life is predesignated. You have to walk the right path which means being there for others.'* Participant A3's mother was his shining example in Christian virtues (and Mary). Participant A3 tells that he is, as his mother, a person who loves people: generous and providing help to others. He regrets not being able to go to church anymore due to his physical condition.<br>(A3.1, A3.2, A3.3)
1b. How does the person retain MiL?	Faith enabled Participant A3 to survive difficult periods in his life. He tells he has come to terms with his past. He prays daily. He stresses that his faith provides him with strength, trust and positivity. He won't share his troubles with others. '*I keep my sorrow to myself. You cannot share it; it wouldn't help. Every person has his own path in life.'* He emphasises he always *'goes on with his daily chores'* when times get rough. *'Don't complain, don't think about it so much.'* Participant A3 arranged a mobility scooter, so he will be able to 'walk' his dog, when his physical condition may deteriorate. He also made last arrangements, to safe his sister work.<br>(A3.1, A3.2, A3.3)
1c. What does he/she expect from the nurse?	Participant A3 doesn't expect nurses to pay attention to MiL and he prefers not to share his sorrow. Participant A3 believes nurses' only task is the physical care. However, Participant A3 is confident with the care he receives. '*You know, you shouldn't bother others with your grief and worries. You just shouldn't ... you know: they have their own family.*

	They are there for their work. They don't have the time to sit and talk. They come to dó something. They help me taking a shower; they dry and rub me in with body lotion. They even dry the shower stall'<br>Furthermore, Participant A3 thinks nurses won't understand, because they don't share religious backgrounds.<br>(A3.1, A3.2, A3.3)
2. Does the nurse recognize the person's MiL (and the way he/she deals with it)?	Although Participant A3 doesn't perceive nurses' recognition, the behaviour of the some nurses shows that they notice and understand the grief and loneliness of Participant A3. They visit Participant A3 during lunchtime.<br>(A3.2, A3.3)
3. How does the nurse respond to the patient (attunement to MiL)?<br><br>a. to the struggle, concern, vulnerability, need or pain of the aged person?<br><br>b. to the strength and resilience of the aged person?	Although Participant A3 doesn't share much with nurses, some nurses try to ask follow-up questions. *'I always tell them that I am fine. And they say: you always say you're fine. And then I respond: complaining doesn't help.'*<br><br>Participant A3 told to some of the nurses about his recent loss. *'I told them about the loss of my last friends… eh… and they sympathised with me but you know: they have their own family. So I keep it as much as possible to myself'* Conversation didn't go any deeper and Participant A3 didn't want to bother the nurses.<br><br>Lunch visits of nurses can be regarded as a response to the perceived loneliness of Participant A3. Some of the nurses even walk the dog, when Participant A3 had a serious flu, which was most important to him.<br><br>Participant A3 tells that one of the nurses had proposed him to go to a (community) dinner in a nearby school and had suggested to ask a few neighbours. Participant A3 enjoyed the evening and was happy with the suggestion of the nurse.<br><br>Participant A3 tells about the temporal personnel: He won't allow them to help his taking a shower. *'I am not going to undress for an unknown person! I just told I did it myself already.'*<br><br>Participant A3 experiences the lunch visits as an opportunity to be a generative person: *'Sometimes when they have a free hour they come to my home. I tell them: come to me. I'll make you tea, coffee, whatever you want. And then they eat their lunch sandwich here and I really enjoy that.'* He daily provides fruit and specific drinks to the nurses because he knows they love it. Participant A3 also buys a personal Christmas gift for them. *'You have to be friendly and interested in them too.'* He highly enjoys the stories of the nurses during daily care.<br>(A3.2, A3.3)
4. Does the care offered do well to the patient?<br><br>a. If yes: what is the consequence?	Yes and no.<br><br>Participant A3 enjoys the lunch visits of well-known nurses. The relationship feels equal and reciprocal to him. Comparable to Participant A3 in his younger years, the nurses need to work to make a

	living. *'I am a working person, just like them.'* He tells about the lunch visits: *'Then you have different conversations. More about what's on their mind. And they say to me: You are just like a mom to us. And then we're joking around.'*<br><br>Participant A3 appreciated nurse's suggestion to go to the dinner and he is currently making plans to go again together with his neighbours.
b. If not: what is the consequence?	The care of temporary staff doesn't feel well. Participant A3 is not cared for as good as normally and he misses the reciprocal contact with the trusted nurses.<br>(A 3.2, A3.3)
Additional remarks	
