# Supplementary material for: Nurse’s attunement to patient’s meaning in life - a qualitative study of experiences of Dutch adults ageing in place
Source: BMC Nurs. 2020 May 18;19:41. doi: 10.1186/s12912-020-00431-z (PMC7236336; doi:10.1186/s12912-020-00431-z)
Supplement: Supplementary file 4 — Additional file 4. Analysis of participant B4 [file 12912_2020_431_MOESM4_ESM.pdf]

Analytical questions	Participant B4 (Age 91-95)
Introduction<br>1. What is at stake for the aged person?	Participant B4 looks younger than his age but his condition is fragile, due to severe heart failure which limits activities. The self-made paintings on the wall are from years ago. Participant B4 is unable to go out and as a result he has no contact with neighbours. During our conversations he repeats himself many times. However, Participant B4 tells that he's content that his *'mind is still working well.'* Although he claims that he is not a talkative person, he shares many stories with me. Participant B4 had a career in a company for beauty products and he loves to tell about that. He also tells about his private life: *'I didn't have an easy life. My partner was untrue. My partner was in jail several times and transmitted me a venereal disease. I was longing for a happy, warm family life but I didn't have that...'* Participant B4 had one son. Participant B4 worked hard and did everything to prevent his son from following the bad example of his parent. His son became *'an honest guy'* and he was proud of him. Unhappily his son died when he was 43 years old. *'I dream of him every night.'* He tells his dream: *'I dream that I am on my bike and he, a little boy, is sitting behind me. I worked during lunch breaks, so I prepared sandwiches for my son and kept them fresh between two plates... and when he went back to school he passed by and waved behind the shop-window and I waved back'*. The relationship with his daughter-in-law is warm. His grandchildren don't visit him. Sometimes Participant B4 calls them. Home nurses come by four times a day. Participant B4 says: *'They come to look after me because I am very old and have nobody. They check on me.'* And he adds *'I am doing fine but the most beautiful thing would be to peacefully close my eyes.'*
a. What are MiL sources for the aged person?	Although life wasn't easy for Participant B4, he is at peace with the way he lived it. MiL sources are:<br>• Happy memories of his son and his work.<br>• His own personality. Participant B4 is a self-conscious person and he is satisfied with what he achieved. He sees himself as a kind person who can interact well with people and as a strong, self-reliant person: *'Sometimes you have to jump into the deep. Life's not a bed of roses. There was nobody to catch me, I all did it by myself…I am strong. That's what life made of me. I am a go-getter. I want to have a good life and most of all: I wanted a good life for my son.'*<br>• The relationship with his daughter-in-law: *'She is a gem. When my son was dying, he asked her to care for me, and she does so very well! That comforts me.'*<br>• Everyday life. *'I take life as it is. I have my puzzle-books, the television. And the girls (home nurses) come by.'*<br>(B4.1, B4.2, B4.3)
1b. How does the person retain MiL?	Participant B4 accepts old age and its limitations. *'That's logical when you are so old.'* He does exercises every day and makes sure he looks good. *'I keep on taking care of myself as I always did.'* Participant B4 relies on himself. He tells he never asks help from others. *'I cope with everything myself. I always say: I am fine'*. However, at night dreams and thoughts keep him awake. Participant B4 tells: *'I don't want to think too much about these things. I turn to my puzzle-books and the*

	television.'<br>(B4.1, B4.2, B4.3)
1c. What does he/she expect from the nurse?	Participant B4 accepts that the nurses visit him four times a day. He explains: *'They have to do that because I am so old.'*<br>He expects them to be polite but he doesn't expect them to have attention for MiL. He thinks nurses are not capable to discuss MiL. *'The girls are so young. They are a bit shy. I have to keep the conversation going. They are not able to discuss such a matter.'*<br>(B4.2)
2. Does the nurse recognize the person's MiL (and the way he/she deals with it)?	Participant B4 thinks that the contact is too short to notice MiL aspects. *'They come to watch if you are not dead. The contact is too short to form an opinion of such a thing (as MiL).'* However, nurses who stay a little longer with Participant B4 possibly notice his loneliness and grief and also his strength.<br>(B4.1, B4.2)
3. How does the nurse respond to the patient (attunement to MiL)?<br><br>a. to the struggle, concern, vulnerability, need or pain of the aged person?<br><br>b. to the strength and resilience of the aged person?	Participant B4 tells that all nurses are very kind and polite towards him.<br><br>Participant B4 tells he doesn't want attention for his worries and sorrow. *'I am a closed person. I don't want to talk about the past with everybody. I am not pitiable!'*<br><br>Participant B4 tells that the home nurses like to come with him. *'The girls always say: it is so cosy with you.'* Participant B4 believes this is a result of his long experience as a manager. *'They can rest here for a little while. I can relate to them, start a light conversation with them, because I worked with people all my life'*<br>(B4.1, B4.2, B4.3)
4. Does the care offered do well to the patient?<br><br>a. If yes: what is the consequence?<br><br>b. If not: what is the consequence?	Yes. Participant B4 says: *'I couldn't wish better.'*<br><br>It feels safe for Participant B4 to know that the nurses are watching over him.<br>Furthermore he appreciates the kind contact with the nurses. It confirms him in his quality to interact with people. *'Of course I enjoy it that they say: it is cosy with you. You know, that's because I worked with people all my life'* and he tells another example from his job.<br><br>(B4.1, B4.2, B4.3)
Additional remarks	
