# Supplementary material for: Nurse’s attunement to patient’s meaning in life - a qualitative study of experiences of Dutch adults ageing in place
Source: BMC Nurs. 2020 May 18;19:41. doi: 10.1186/s12912-020-00431-z (PMC7236336; doi:10.1186/s12912-020-00431-z)
Supplement: Supplementary file 5 — Additional file 5. Analysis of participant C6 [file 12912_2020_431_MOESM5_ESM.pdf]

Analytical questions	Participant C6 (Age 81-85)
Introduction<br>1. What is at stake for the aged person?<br><br><br><br><br><br><br><br><br><br><br><br><br><br><br>a. What are MiL sources for the aged person?	Participant C6 and his partner moved from Suriname to the Netherlands in the sixties. Participant C6 worked for labour subcontractors as a welder. The couple had three children. Two of them died very young. Participant C6's partner died, only 49. He shows me a photo of an elegant young person. Participant C6 tells he doesn't think a lot about his losses. *'Those are things which have to happen. Everything is written already. Our lives are predesignated. And therefore you can live as you wish. Nobody has to tell another how to live. I am free.'* Participant C6's son lives in a city far from Rotterdam. He calls now and then but they don't see each other frequently. A friend comes by now and then to cook and do the shopping for Participant C6. Participant C6 has severe diabetes. He used to control and inject himself but due to deteriorating vision he is unable to do this anymore. Therefore nurses visit him four times a day. His foot has a dirty wound, which is dressed by the home nurses. Participant C6 suffers from pain in his back.<br><br>Participant C6's MiL sources are:<br>• Going out and meeting friends. At the time of the first interview, pain limits Participant C6's mobility. He says '*When I am in pain, that day is nothing for me. I am cursing in Surinam… I stay inside and talk to nobody… Not being able to go out, is the worst thing that can happen to me.'* Between the first and second interview one of his toes was amputated and pain in his back diminished. He is going out again and meeting with friends. *'A good day is when I am talking with other people about what happened long time ago and laughing about that.'* Participant C6 is proud to have friends from all over the world.<br>• Social engagement. Participant C6 talks a lot about society, history, especially from Suriname, and actual news. He is very aware of differences among people (Dutch-Surinam, men-women). He emphasises values as solidarity and equality. '*We are all people and we have to help each other.'*<br>• Music. Participant C6 listens to music all day. He used to play guitar but he gave it away to a niece. *'We, Latin-Americans, have music from Cuba and Jamaica. We are rhythmic people. That's great to play!'*<br>(C6.1, C6.2, C6.3)
1b. How does the person retain MiL?	Participant C6 perceives himself as a sociable person. *'I talk with everybody.'* He is used to tell stories and making a lot of jokes, also during our conversations. He follows the news on the radio. *'When you hear the news you can talk with friends about it.'* Participant C6 used to be a person who did everything himself. *'I don't like to ask people things.'* He adapts his habits to his actual condition, for instance, in order to walk bigger distances, Participant C6 walks with the support of a bike. He is pondering to learn how to use different instruments so he can manage his diabetes himself again. In the third interview Participant C6 tells he is considering going to a nursing home.<br>(C6.2, C6.3)
1c. What does he/she expect from the nurse?	Participant C6 expects from nurses that they keep an *'appropriate distance between man and woman'*.

	Furthermore he expects nurses to do what he cannot do himself anymore and watch a bit over him. He expects them to perform their work according to *'normal standards'* and ads that he cannot assess this, because it is not his profession.<br>(C6.1, C6.2, C6.3)
2. Does the nurse recognize the person's MiL (and the way he/she deals with it)?	Participant C6 experiences nurses as attentive. Participant C6 tells an example of an experience when his glucose was low. The nurse who visited him immediately understood the situation. Furthermore Participant C6 tells stories about nurses who ask him if they can do anything for him. Participant C6 thinks they do this because they notice that he is not able to do it himself anymore.<br>Whether nurses perceive other aspects of his MiL is not clear from Participant C6's perspective. Participant C6 answers: *'I cannot know what they perceive.'*<br>(C6.1, C6.2, C6.3)
3. How does the nurse respond to the patient (attunement to MiL)?<br><br>a. to the struggle, concern, vulnerability, need or pain of the aged person?<br><br>b. to the strength and resilience of the aged person?	Although Participant C6 would rather be independent of home nurses, he appreciates the care they provide.<br><br>Participant C6 says: *'They do their work well: fast and well … When they dress my wound they are very careful not to hurt me. That's humane. And they bind my slippers onto my feet, so bacteria don't get into my wound, because I cannot see it. They are caring.'*<br><br>With some nurses of permanent staff relations are closer. They help Participant C6 with administration and mail which is difficult for him to do himself, due to limited vision and mobility. He is grateful for that. *'Some people say that people are malicious nowadays… but I can rely on those trusted nurses. They do it out of love for me…Those things are solidarity, no matter who you are.'*<br><br>The nurse who noticed that Participant C6 had a hypo reacted adequately: she assisted him to a chair and made some lemonade for him.<br><br>Participant C6 perceives himself as a sociable person. He enjoys the relationship with permanent staff members. *'They are young and free, some are Dutch, one is Antillean and there's a Cape Verdean. I believe I had luck with this group. We have a chat and some ask if they can do something for me. I appreciate that.'*<br><br>There is one special nurse. She shares Participant C6's love for music. '*When she comes she always asks: shall we put the volume a bit higher? Yes, with her… we laugh together and enjoy the music.'*<br><br>Participant C6 loves his freedom and independence. Sometimes it is difficult for him to be at home on time for the nursing visits. He tells that nurses call him when he is not there. They don't complain. They change their schedule and visit him later.<br>(C6.1, C6.2, C6.3)
4. Does the care offered	Yes, the offered care feels good, although visits limit his freedom.

do well to the patient?<br><br>a. If yes: what is the consequence?<br><br><br><br><br><br><br><br><br>b. If not: what is the consequence?	 <br>The fact that nurses look over him and notice when something is wrong makes Participant C6 feels secure.<br><br>Because he doesn't like to ask anything from another, Participant C6 appreciates that nurses notice things which are difficult to accomplish for him and offer him help.<br><br>The relationship with nurses and the personalised, skilled care are of great value to Participant C6. *'That's humaneness!'*<br><br>Although Participant C6 appreciates the home nurses highly, the four daily visits limit his freedom: *'My life organisation has changed. I cannot go where and whenever I want, because I have to be home for them.'* (C6.1, C6.2, C6.3)
Additional remarks	
