# Supplementary material for: Nurse’s attunement to patient’s meaning in life - a qualitative study of experiences of Dutch adults ageing in place
Source: BMC Nurs. 2020 May 18;19:41. doi: 10.1186/s12912-020-00431-z (PMC7236336; doi:10.1186/s12912-020-00431-z)
Supplement: Supplementary file 6 — Additional file 6. Analysis of participant B5 [file 12912_2020_431_MOESM6_ESM.pdf]

<table>
<tr><th>Analytical questions</th><th>Participant B5 (Age 71-75)</th></tr>
<tr><td>Introduction
1. What is at stake for the aged person?

a. What are MiL sources for the aged person?</td><td>Participant B5 was a successful entrepreneur until his stroke, two years ago. He and his partner had to be persistent to be admitted to intensive rehabilitation therapy but succeeded. Participant B5 has reached a high level of independence after his stroke, due to the specialised therapies and intensive exercise. Home nurses visit the couple once a day to assist Participant B5 with bathing and grooming. Although he is proud of what he accomplished, the - partial - independence annoys him, for instance not being allowed to drive a car or to play tennis anymore. He keeps searching for, and achieving, further improvements. His partner is a great support for Participant B5, both physically and mentally. Participant B5 perceives himself as a society-critical person. He is worried about recent transitions in elderly care and the future of young people. The couple has a full agenda: they are active community members in the neighbourhood where they live.

Participant B5 lives a dynamic life. Sources for Mil are:
Looking back on a life which he regards as successful: 'I had a tremendous life. I had several companies, saw the whole world. I had the luck to meet good people.'
Independence.
Being there for others in the community, especially those in vulnerable conditions. 'I hate injustice. I hate indifferent, incompetent people… We want to be there for others, to make the community more liveable.'
His partner. Participant B5 regrets that most attention went to his health last years. 'MiL is for me: doing things together with [name partner].'
(B5.1, B5.2, B5.3)</td></tr>
<tr><td>1b. How does the person retain MiL?</td><td>Participant B5 exercises every day and does as much as he can by himself, also in daily care. He tells he is open to others, especially when they are in trouble. Participant B5 experiences himself as a doer and a fighter, who can accomplish things because of his character and experience. 'I am a fighter… I want to show that you can accomplish a lot by will power.'
Furthermore he enjoys life, his achievements of the past and his present life with his partner.
(B5.1, B5.2, B5.3)</td></tr>
<tr><td>1c. What does he/she expect from the nurse?</td><td>Because he has an active life it is very important for Participant B5 that nurses come on time. He expects them to do their work with pleasure. 'If they enter the house with pleasure, we have pleasure in them being here.'
Participant B5 thinks 'nurses don't have the psychological knowledge to discuss MiL.'
(B5.1, B5.2, B5.3)</td></tr>
<tr><td>2. Does the nurse recognize the person's MiL (and the way he/she deals with it)?</td><td>Since organisational transitions started, nurses are occupied with organisational troubles. Participant B5 notices that this limits their space to be open to patients. 'The nurses are being jerked around. Those changes in the organisation are an excuse for other procedures here. And [name nurse] has to explain all that to us, in her free time. But that has nothing to do with us. That's not our business. We listen to them but</td></tr>
</table>

	it distracts from what they come for. But most of all, it limits the pleasure they have in their work. And that is important to us too.'<br>(B5.2, B5.3)
3. How does the nurse respond to the patient (attunement to MiL)?<br><br>a. to the struggle, concern, vulnerability, need or pain of the aged person?<br><br>b. to the strength and resilience of the aged person?	Participant B5 mentions that there is a big difference between nurses. He and his partner maintain cordial relations with the permanent staff but he complains about the temporal staff. As an entrepreneur he doesn't understand why management did replace nurses, while the team was functioning well.<br><br>Nurses seldom come on time. When Participant B5 had a hospital appointment they were more than an hour late. '*I do as much as I can by myself. I had to be in the hospital on time. The taxi will not wait. It intrudes in my life when they are too late. I was there sitting and waiting, and they even didn't call to say they were late.'* It was not the first time.<br><br>The uncertain time of visits of the nurses is also inconvenient for his partner: she feels she has to adapt to nurses' schedule, unable to plan when to use the bathroom and get dressed.<br><br>In the second interview Participant B5 mentions a special nurse of the permanent staff. In an unexpected difficult situation, she came immediately, in her free time, and did everything that was needed. *'These few are toppers. When you call them that something is wrong, they come immediately. No bullshit: Just being there when they are needed.'*<br><br>Participant B5 had to explain to new and temporal staff that he wants to do himself as much as he can, to regain his capabilities. *'And then they open the bathroom door many times: "can I wash your back?" (irritated) No, I do it myself, don't you understand!? Some of them were terrible. I prohibited some of them to come again.'*<br><br>Participant B5 and his partner feel the pressure of permanent staff, due to organisational change, and they serve as an outlet for them. *'You just feel that when you know each other well… And sometimes they tell a little bit about that… And we listen. I understand they need a way to vent their feelings. I hope they feel relieved afterwards. That is no problem for us '*<br>(B5.2, B5.3)
4. Does the care offered do well to the patient?<br><br>a. If yes: what is the consequence?<br><br>b. If not: what is the consequence?	Yes and no<br><br>Participant B5 enjoys the contact with permanent staff. When they do their job with pleasure it influences his day. They provide the care well and if they are on time he can carry on in his active life and mean something for others. A few nurses are special ones: he knows he can count on them.<br><br>Although Participant B5 and his partner experience that they are '*a sounding board for them*', he believes that nurses' attention is

	distracted from their principal focus -the relation with patients- and their pleasure in work, when nurses are occupied with organisational problems.<br><br>Furthermore, Participant B5 is limited in the life he wants to live, when nurses don't respect his independence, or don't come on time. However, he doesn't blame the nurses for this but the healthcare organisation. *'I was used to care independently for myself and my partner all my life. And when they don't come on time I lose part of my life. We don't blame those nurses we know. It's taken away by the policy of an organisation. It makes me feel curtailed...'*<br>(B5.2, B5.3)
Additional remarks	Participant B5 gladly wanted to participate in the study. He hoped it contributes to improvements in healthcare.
