# Supplementary material for: Nurse’s attunement to patient’s meaning in life - a qualitative study of experiences of Dutch adults ageing in place
Source: BMC Nurs. 2020 May 18;19:41. doi: 10.1186/s12912-020-00431-z (PMC7236336; doi:10.1186/s12912-020-00431-z)
Supplement: Supplementary file 7 — Additional file 7. Analysis of participant C5 [file 12912_2020_431_MOESM7_ESM.pdf]

Analytical questions	Participant C5 (Age 66-70)
Introduction<br>1. What is at stake for the aged person?	Participant C5 grew up in Turkey in the mountains. Her partner went to the Netherlands as a labour immigrant and he followed several years later. The couple had five children and thirteen grandchildren. Four children live nearby. The children and grandchildren share the care for their parent, as they had done before, together with Participant C5, for their other parent before he died. They maintain warm relationships. Participant C5 suffers from terminally metastased cancer. He had several operations and treatments, both in Turkey and the Netherlands. Pain and exhaustion limit activities. However, the family hopes there will be time to enjoy another summer in the beloved mountains in Turkey, where the climate is beneficial for him. Home nurses visit Participant C5 once a day to dress a radiation wound on his shoulder which is very painful. Participant C5's Dutch is limited but he has a very expressive face. Her children assist him during our conversation.
a. What are MiL sources for the aged person?	Participant C5's MiL sources are:<br>• Faith (Islam). In the room is a picture of Mekka. He tells he went to Hadj when he was younger. Living as a good Moslim is important to him. He adds: *'Being a good Muslim is not slaughtering other people. Good Muslims help others and bring about peace... It is not good to focus on differences. All people are brothers: black, Dutch, Turks…'*<br>• His children and grandchildren. Participant C5 is happy being surrounded by family. *'I am happy to have five children who all love me. They are always there. Whenever I feel sick they comfort me. I pray to Allah that all people may have such good children.'*<br>• Nature. Participant C5 loves flowers, plants, birds. He loved gardening. His current condition limits this but he still enjoys being outside or watching the garden from inside.<br>(C5.1)
1b. How does the person retain MiL?	Living as a good Muslim. Praying provides him with peace in mind. For Participant C5 being a good Muslim also involves being kind to others. He always has been a welcoming person and still is. Besides family, neighbours visit him frequently. He donates to charity, like the cancer fund. Participant C5 loves to look out of the window on the gardens below. He also liked to feed the birds on his balcony, although his daughter asked him not to do so because of the dirt they produced. When children and grandchildren take him out in a wheelchair he enjoys this highly.<br>(C5.1)
1c. What does he/she expect from the nurse?	Participant C5 and his children) mainly expect skilled physical care of the nurses. Participant C5's children emphasise that care on other domains than the physical is unwanted; they care for it all.<br>(C5.1)
2. Does the nurse recognize the person's MiL (and the way he/she deals with it)?	Participant C5 and his children notice that the nurses of permanent staff know how to behave towards him. They don't think that nurses recognise MiL, because they don't ask and Participant C5 doesn't tell. It is private.<br>(C5.1)

3. How does the nurse respond to the patient (attunement to MiL)?<br>a. to the struggle, concern, vulnerability, need or pain of the aged person?<br><br><br>b. to the strength and resilience of the aged person?	Depending on the nurse. Participant C5 and his children are more positive about permanent than temporal staff.<br><br>The dressing of the wound can be very painful. Permanent staff members know exactly how to do this in order to let it be bearable for Participant C5. However, he also had bad experiences with nurses who were not skilled to dress the wound *'It was so painful! I was screaming out, crying and trembling.'*<br><br>The family system is strong: After the bad experiences the family consulted the family doctor. Now there is an appointment with the homecare organisation that only experienced nurses are allowed to dress the wound.<br>Participant C5 stresses several times that the nurses of permanent staff are *'all good women, very sweet'* and he smiles. He has two special ones. They have known him for several years. They are cheerful. They take time to chat, have a drink and make a little joke with him. The family invited them on the engagement party of Participant C5's granddaughter.<br>(C5.1)
4. Does the care offered do well to the patient?<br><br>a. If yes: what is the consequence?<br><br><br>b. If not: what is the consequence?	Yes, nowadays it does, although Participant C5 had some bad experiences in the past.<br><br>If nurses know how to treat the wound and do it carefully, it doesn't hurt and Participant C5 saves energy for the rest of the day. Participant C5 feels cheered up when the two favourite nurses visit him.<br><br>When the dressing of the wound was not done properly it caused a lot of pain. He was stressed by the situation and couldn't sleep or eat anymore. Participant C5's daughter tells that Participant C5 lost 15 kilos during that period.<br>(C5.1)
Additional remarks	Participant C5 was interviewd one time. His children stressed that due to his prognosis they didn't want more interviews: they rather saved Participant C5's energy for happy moments. Participant C5 agreed.
